# Meta-analysis of the clinical efficacy of laparoscopic appendectomy in the treatment of acute appendicitis

**DOI:** 10.1186/s13017-022-00431-1

**Published:** 2022-05-26

**Authors:** Guangzhe Zhang, Bo Wu

**Affiliations:** Department of Anorectal Surgery, The First Hospital of China Medical University, Shenyang, 110001 Liaoning China

**Keywords:** Appendicitis, Laparoscope, Open appendectomy, Meta-analysis

## Abstract

**Background:**

This paper compares the postoperative recovery of patients with acute appendicitis (AA) after laparoscopic appendectomy (LA) and open appendectomy (OA), aiming to determine the optimal diagnosis and treatment plan for appendectomy.

**Methods:**

Related literature was retrieved from PubMed, Web of Science, Embase, CNKI and Wanfang databases. Articles on LA and OA for AA published between 2010 and 2021 were selected to extract data. Besides, Stata16.0 was used for meta-analysis.

**Results:**

A total of 777 articles were retrieved, and 16 of them were finally selected. Totally, 1251 patients underwent LA, while 898 patients received OA. According to the results of meta-analysis, LA was associated with lower incidence of adverse reactions [OR = 0.257, 95% CI (0.162, 0.408), *P* < 0.001], shorter operation time (SMD = − 1.802, 95% CI − 2.435, − 1.169; *P* < 0.001) and hospitalization (SMD = − 1.184, 95% CI − 1.512, − 0.856; *P* < 0.001). In addition, compared with the OA group, LA was found with less intraoperative blood loss (SMD = − 3.650, 95% CI − 5.088, − 2.212; *P* < 0.001) and shorter recovery time of gastrointestinal function (SMD = − 3.010, 95% CI − 3.816, − 2.203; *P* < 0.001). Aside from all these, the counts of leukocyte (SMD = − 0.432, 95% CI: − 0.775, − 0.089; *P* = 0.013), neutrophil (SMD = − 1.346, 95% CI − 2.560, − 0.133; *P* = 0.030), and C-reactive protein (SMD = − 2.391, 95% CI − 3.901, − 0.882; *P* = 0.002) all decreased in a significant manner after LA.

**Conclusion:**

Compared with OA, LA boasts the advantages of less adverse reactions, shorter operation time and hospitalization, fewer complications, and lower inflammatory response, evidencing its safety and feasibility of applying in the treatment of AA.

## Introduction

Acute appendicitis (AA) constitutes a common cause of acute abdominal pain worldwide. It is expected to attack a person with a risk of 7–8% during his lifetime [1], habitually setting in late childhood or early adulthood [2]. Typical symptoms of AA involve periumbilical pain migrating to the right lower quadrant or right iliac fossa pain, also accompanied by rebound pain, fever, nausea, or vomiting [3]. In laboratory tests, changes in the number of leukocytes and increases of C-reactive protein are common in AA patients. Abdominal ultrasound, computerized tomography scanning or magnetic resonance imaging is usually used for its diagnosis [4]. Appendectomy is the standard treatment for AA and can be classified as open (OA) or laparoscopic (LA). LA was first brought up by Kurt semm in 1983, and since then, numerous studies have focused on the comparison of LA with conventional OA [5]. For adults, LA usually means less postoperative pain, faster recovery and fewer surgical complications. Therefore, LA has replaced OA in many centers, with an advantage in hospitalization duration and surgical wound complications [6]. It is no exaggeration that LA has emerged as the gold standard for the treatment of suspected simple appendicitis. A meta-analysis by Athanasiou et al. found LA achieved a more significant improvement in the incidence of concurrent appendicitis in adults compared with OA [7]. However, no systematic study comparing the clinical effects of LA and OA in the treatment of AA has been reported as of now. Therefore, the purpose of this paper is to comprehensively evaluate associated research on recovery and clinical efficacy, recovery and postoperative laboratory parameters after LA and OA surgery, thus determining the optimal approach of appendectomy.

## Methods

### Literature retrieval

Electronic databases such as PubMed, Web of Science and Embase, China National Knowledge Infrastructure, and Wanfang were searched for literature published between 2010 and 2021. The keywords were: “laparoscopic/laparoscopy” AND “open appendicectomy/appendicectomy/open approach” AND “appendicitis”.

### Screening criteria

Inclusion criteria: (1) Study subjects: AA patients undergoing LA and OA, without age limit; (2) Intervention: LA and OA; (3) Outcome measures: at least any of the followings: incidence of adverse reactions after treatment, postoperative white blood cell count (WBC), postoperative neutrophil count (NEUT), postoperative C-reactive protein (CPR), operation time, intraoperative blood loss; recovery time of gastrointestinal function, hospitalization duration; (4)Study design: randomized controlled trials (RCTs) or case–control studies.

Exclusion Criteria: (1) Studies without traceable results; (2) reviews, duplicate studies, animal experiments.

### Data extraction

Research selection was independently performed by two evaluators. Should any disagreement arises, a third evaluator would be introduced for joint consultation or the two discussed with each other to achieve consensus. The following data were extracted from the selected articles: title, country, study design, baseline characteristics of participants and outcome measures.

### Statistical analysis

Data analysis was performed using Stata16.0 software. The heterogeneity of all studies was tested by means of *Q* test and I^2^ statistic. When *P* < 0.05 and I^2^ > 50%, the random-effects model would be used for meta-analysis; otherwise, the fixed-effects model would be adopted. Odds ratios (ORs) or standardized mean differences (SMDs) were calculated with 95% confidence intervals (CIs) for dichotomous and continuous outcomes. Funnel plots were used to detect publication bias, and sensitivity analyses were performed to further confirm the stability of the overall effect. *P* < 0.05 was considered statistically significant.

## Results

### Results of literature retrieval

**Fig. 1:**
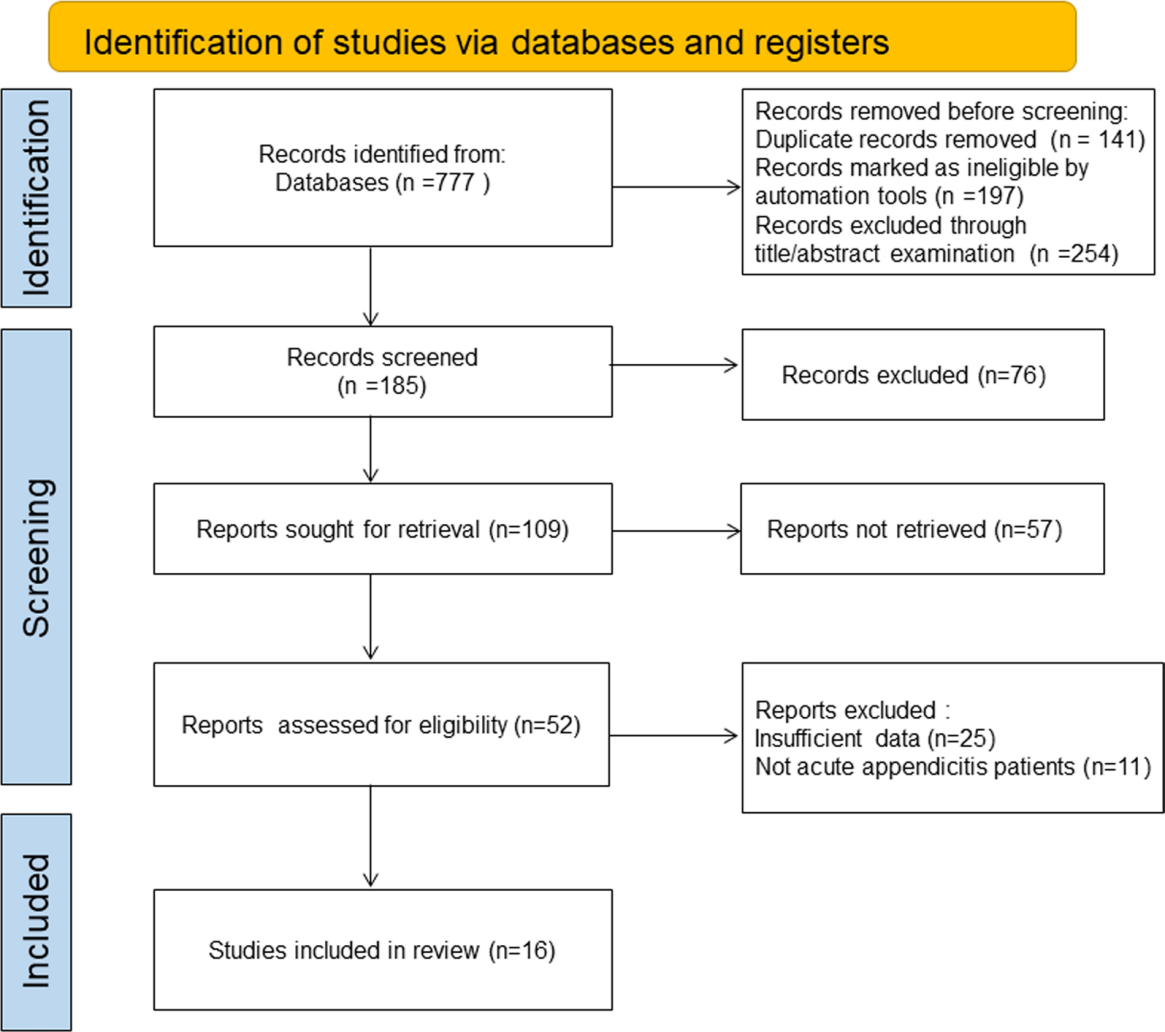
Flow chart of literature screening

Initially 777 studies were included based on the results of electronic database searching. Finally 16 studies were considered qualified after removing 141 repeated articles, 197 unqualified articles marked by automated tools, 254 articles through examining their titles and abstracts, 76 with unqualified data, 57 duplicate reports, 25 insufficient in data, 11 with unsuitable study subjects[8–23] (Fig. 1).

All 16 studies were both RCTs and retrospective studies with 10 Chinese articles and 6 English articles. In total, 2149 patients were included, with 1251 patients receiving LA and 898 patients receiving OA. In the included articles, subjects varied in age from 9 to 50 years old and sample sizes ranged from 37 to 531. The characteristics of each included study are summarized in Table 1.

**Table1:** The basic characteristics of included literature

Study	Year	Sample time (year.month)	Cases LA/ OA	Age (years)		Sex (male/female)		Study design	Outcome measures
				LA group	OA group	LA group	OA group		
Xu Huadong	2021	2019.05–2020.10	41/41	54.37 ± 2.56	53.25 ± 2.54	27/14	28/13	RCT	①②③④⑤⑥⑦⑧
Yuan Bo	2019	2005.01–2015.12	422/109	40.5 ± 16.1	41.3 ± 15.5	272/150	70/39	Retrospective	①④⑤⑥⑦⑧
Yi Zhengguo	2021	2016.01–2019.01	40/40	46.64 ± 5.47	46.52 ± 5.39	27/13	28/12	RCT	①③④⑤⑥⑦⑧
Yang Yuetao	2014	2011.06–2013.06	36/36	50.6 ± 7.4	50.6 ± 7.4	22/14	23/13	RCT	①⑤⑥⑦⑧
Gong Wei	2018	2011.01–2015.12	57/45	9.39 ± 2.90	9.24 ± 2.43	33/24	26/19	Retrospective	①⑤⑥⑦⑧
Shen Zhenghai	2016	2010.09–2015.09	115/138	39.9 ± 14.96	38.03 ± 15.62	70/45	79/59	Retrospective	①②⑤⑦⑧
Li Yongchao	2014	2010.01–2011.02	35/42	41.3 ± 17.3	44.8 ± 16.5	17/18	20/22	Retrospective	①②⑤⑦⑧
Liu Wenbin	2020	2017.01–2019.12	28/28	36.64 ± 3.71	36.58 ± 3.65	16/12	15/13	RCT	①④⑤⑥⑦⑧
Le Hao	2020	2016.09–2018.12	43/43	43.15 ± 4.78	43.10 ± 4.83	21/22	20/23	RCT	①②③④⑤⑥⑦⑧
Wu Ji	2014	2010.03–2013.12	62/54	36.5 ± 5.82	36.8 ± 6.26	31/31	27/27	RCT	①⑥⑦⑧
TomoyaTakami	2020	2011.01–2017.12	90/89	50.13 ± 25.84	50.17 ± 22.77	62/28	56/33	Retrospective	①③④⑤⑥⑦⑧
VincenzoMinutolo	2014	2008.05–2012.05	139/91	12–73	12–48	62/77	43/48	Retrospective	①②⑤⑦⑧
SaeedKargar	2011	2008.04–2009.04	50/50	26.94 ± 9.51	25.36 ± 8.92	23/27	28/22	RCT	①⑤
Ching-ChungTsai	2012	2000.01–2004.11	20/32	9.6 ± 3.6	9.4 ± 3.3	12/8	21/11	Retrospective	①②③④⑤⑧
AliKocataş	2013	NP	50/46	27.4 ± 18.5	28.2 ± 21.2	27/23	42/4	RCT	①
TatyanCLArke	2011	1997.01–2001.12	23/14	19–60	18–50	15/8	9/5	RCT	

LA: laparoscopic appendectomy; OA: open appendectomy; RCT: randomized controlled trial; NR: Not reported; ①: Adverse effects rate; ②: White blood cell count after treatment; ③: Neutrophil count after treatment;④: C-reactive protein after treatment; ⑤: Operation time; ⑥: Intraoperative blood loss volume; ⑦: Gastrointestinal function recovery time; ⑧: Hospital stay. OA, open appendectomy; LA, laparoscopic appendectomy

### Comparison of clinical efficacy indicators

**Fig. 2:**
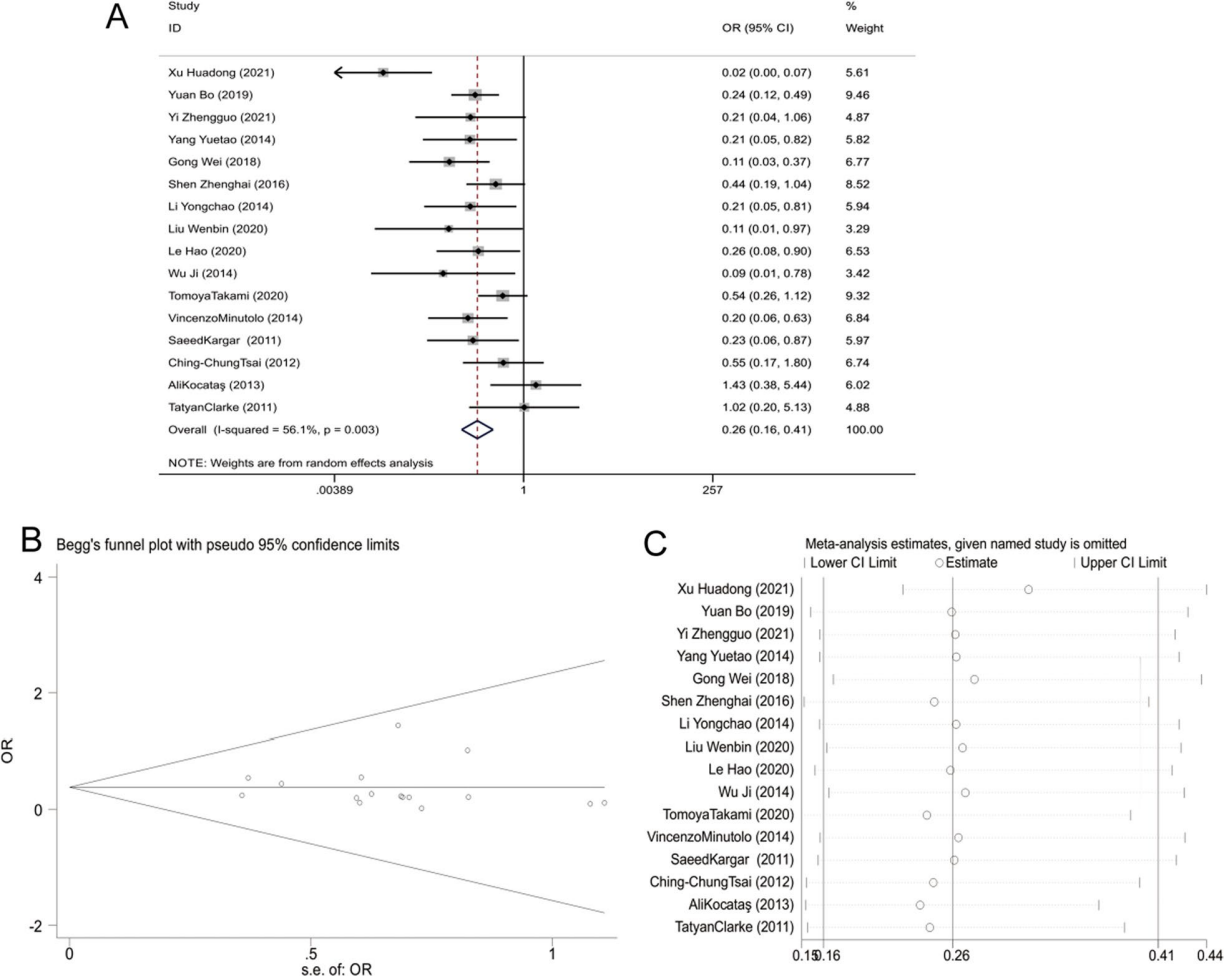
Meta-analysis of the incidence of adverse reactions after appendectomy. **A** Forest plot comparing the incidence of postoperative adverse reactions between LA and OA in patients with acute appendicitis; **B** Funnel plot assessing the potential publication bias in studies discussing the incidence of adverse reactions; **C** Sensitivity analysis of the incidence of adverse reactions. OA, open appendectomy; LA, laparoscopic appendectomy.

Sixteen studies compared the incidence of adverse reactions between the two groups. Moderate heterogeneity among studies (I^2^ = 56.1%, *P* = 0.003) was observed, enabling the adoption of random-effects model to combine the effect size. In accordance with the results, we could find that the incidence rate of postoperative adverse reactions in the LA group was significantly lower than that in the OA group [OR = 0.257, 95% CI (0.162, 0.408), *P* < 0.001, Fig. 2A]. No significant bias in the incidence of adverse reactions was identified in the funnel plot for publication bias detection (Fig. 2B). Besides, the sensitivity analysis displayed little change in the combined result after removing the included studies one by one, indicating comparatively strong stability of the result of meta-analysis (Fig. 2C).

### Comparison of postoperative recovery indicators

**Fig. 3:**
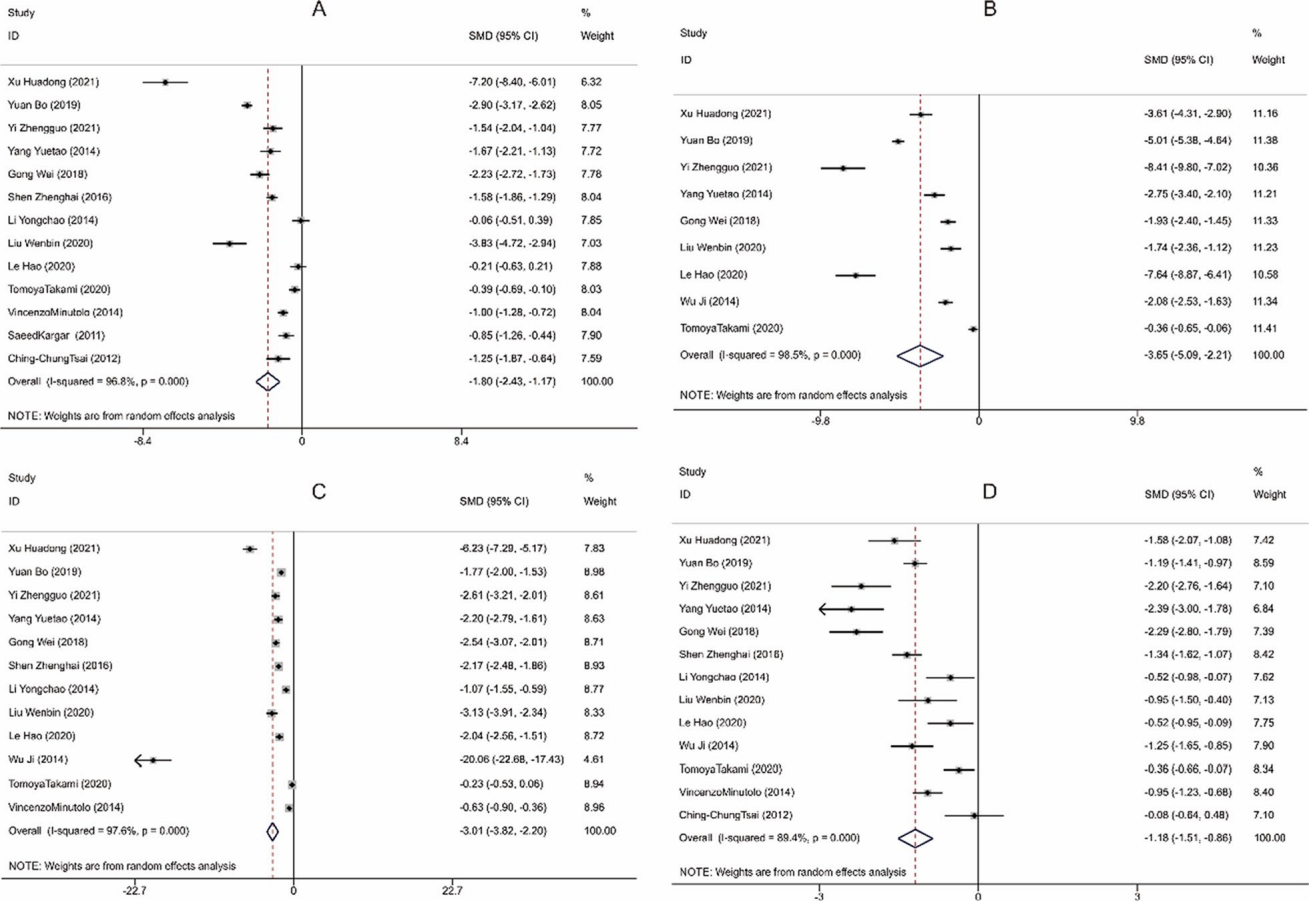
Forest plots comparing the recovery of patients with acute appendicitis after LA and OA. **A** Operative time; **B** Intraoperative blood loss; **C** Gastrointestinal recovery time; **D** Hospital stay. OA, open appendectomy; LA, laparoscopic appendectomy

**Fig. 4:**
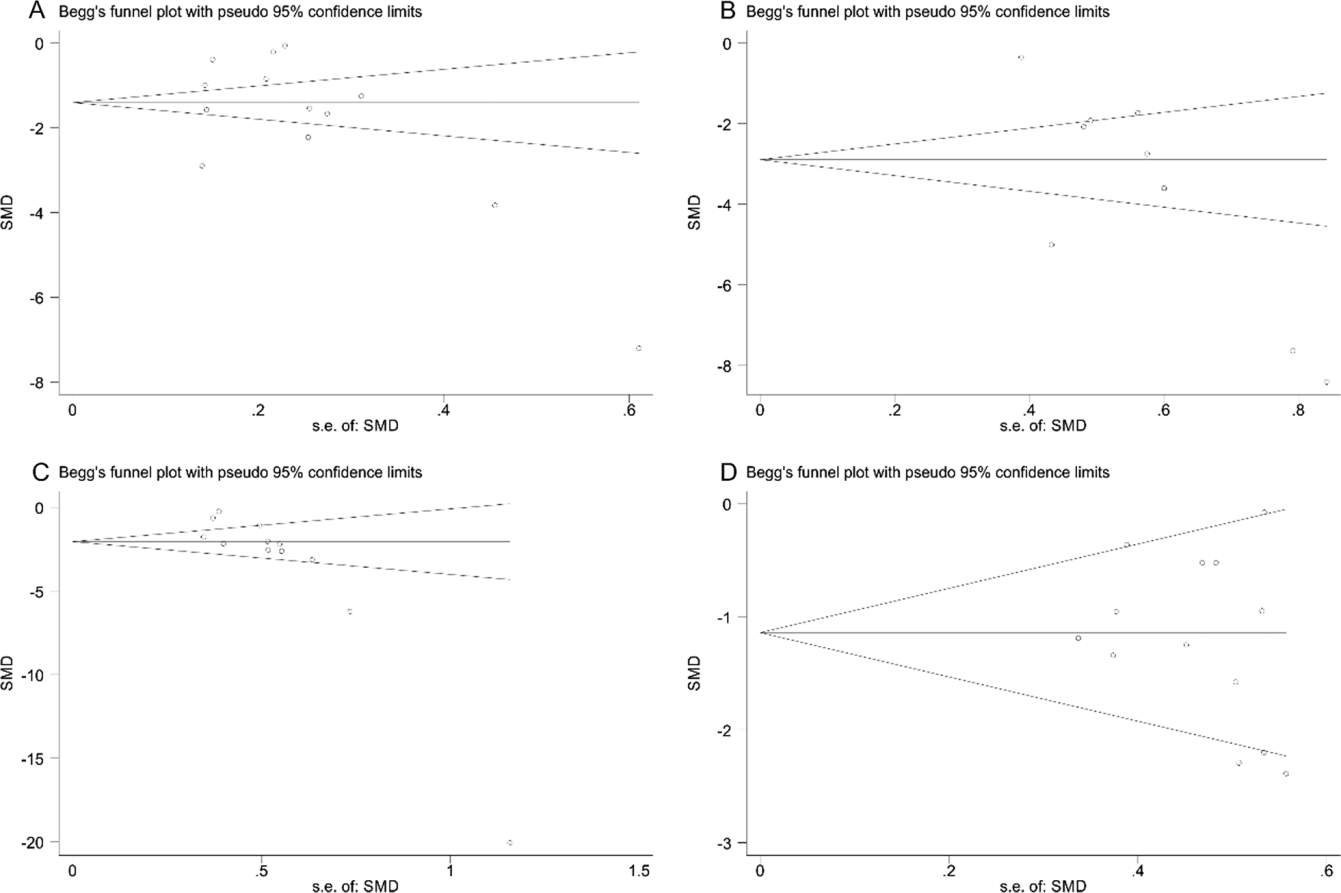
Funnel plots of operating time (**A**), intraoperative blood loss (**B**), gastrointestinal recovery time (**C**), hospital stay (**D**)

**Fig. 5:**
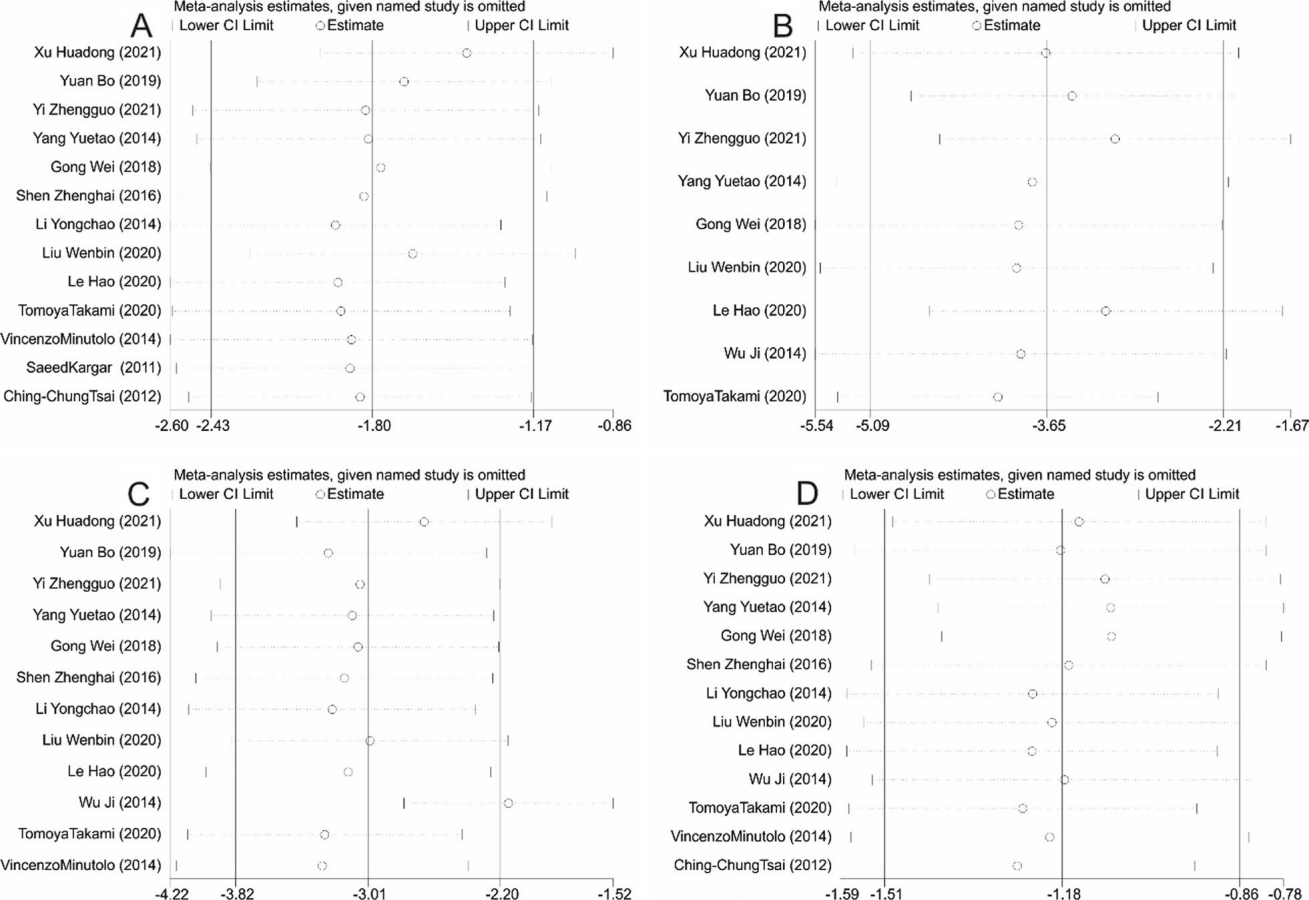
Sensitivity analyses of operating time (**A**), intraoperative blood loss (**B**), gastrointestinal recovery time (**C**), hospital stay (**D**)

Thirteen studies reported the operative time, 9 studies mentioned the intraoperative blood loss, 12 studies compared the postoperative gastrointestinal recovery time, and 13 studies involved the length of hospital stay for patients in the LA and OA groups. The studies included demonstrated significant heterogeneity (I^2^ > 50%, *P* < 0.001) and were thus analyzed using random-effects model. By analyzing the above indicators, it was found that LA performed better than OA. To be specific, LA was associated with apparently shorter operation time [SMD = − 1.802, 95%CI(− 2.435, − 1.169), *P* < 0.001, Fig. 3A], significantly less intraoperative blood loss [SMD = -3.650, 95% CI (− 5.088, − 2.212), *P* < 0.001, Fig. 3B], quicker recovery of gastrointestinal function [SMD = − 3.010, 95% CI (− 3.816, − 2.203), *P* < 0.001, Fig. 3C], and much shorter period of hospitalization [SMD = − 1.184, 95% CI (− 1.512, − 0.856), *P* < 0.001, Fig. 3D]. The funnel plot of publication bias detection showed no significant bias in the operation time, intraoperative blood loss, gastrointestinal function recovery time and hospital stay of the included studies (Fig. 4A–D). Sensitivity analysis further proved the new combined results only had little change from the combined results before exclusion, suggesting low sensitivity and credibility of the results of meta-analysis (Fig. 5A–D).

### Comparison of postoperative laboratory parameters

**Fig. 6:**
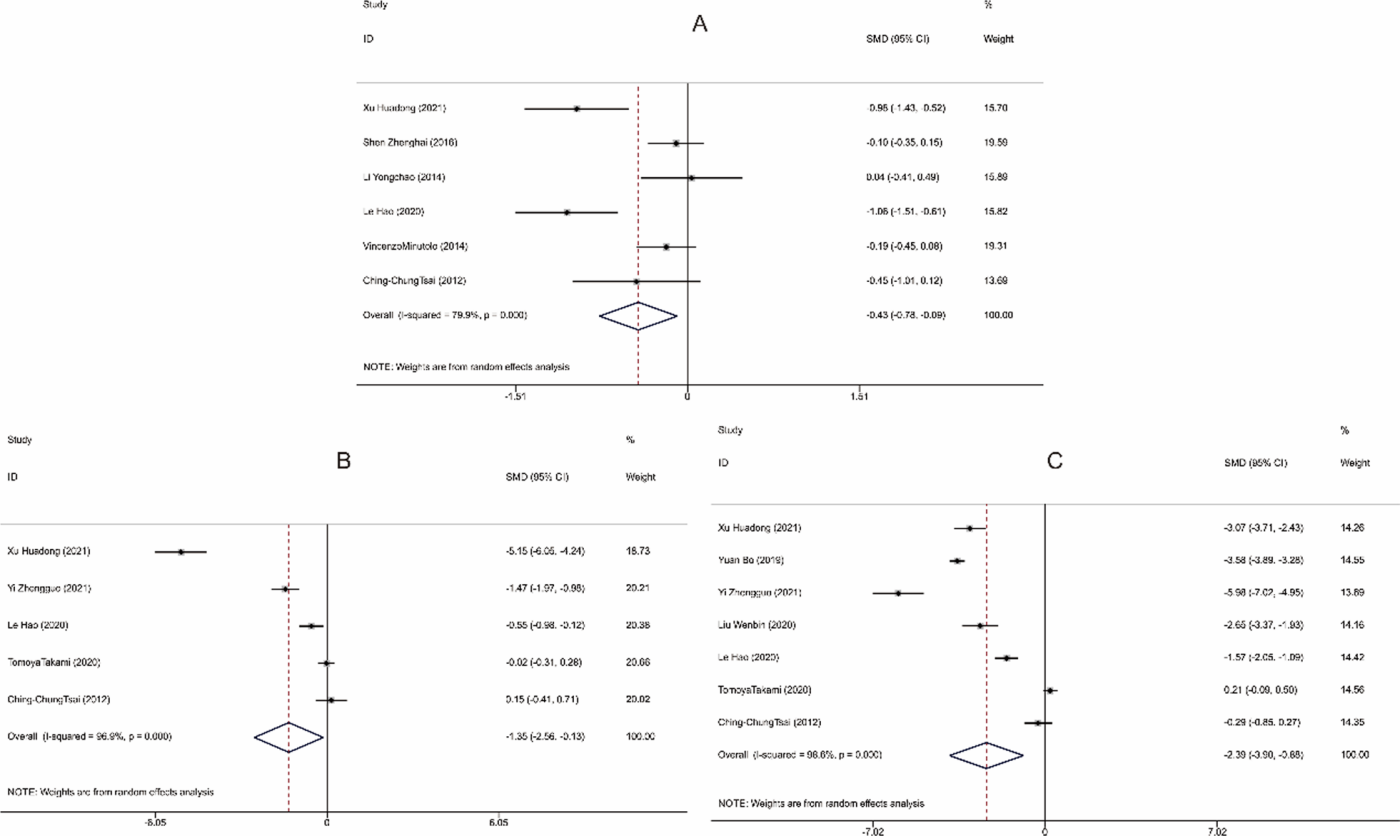
Forest plots comparing postoperative white blood cell count **A**, postoperative neutrophil level **B**, and postoperative C-reactive protein level **C** in patients with acute appendicitis after LA and OA. OA, open appendectomy; LA, laparoscopic appendectomy

**Fig. 7:**
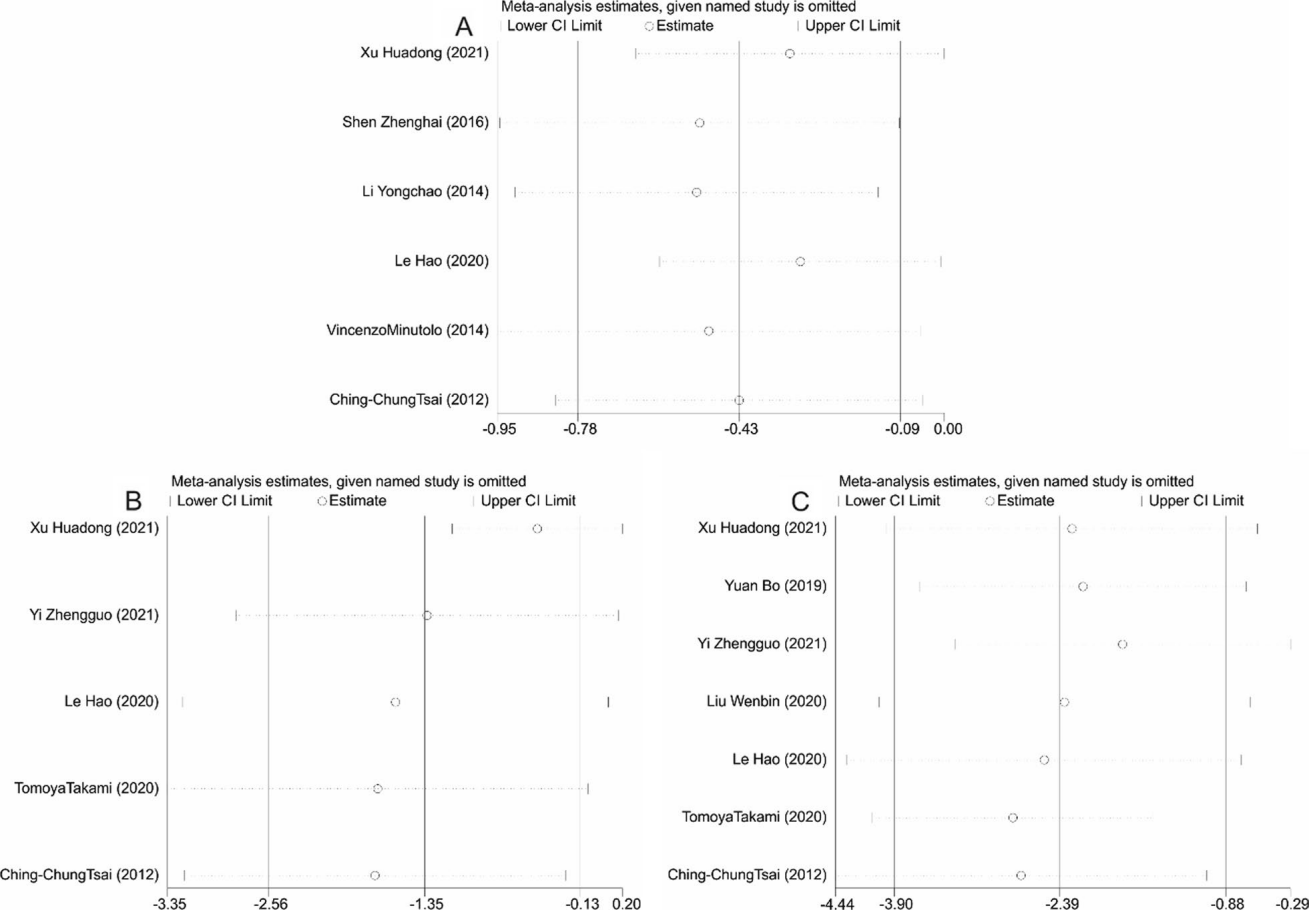
Sensitivity analyses of postoperative white blood cell count **A**, postoperative neutrophil **B** and postoperative C-reactive protein **C** level in patients with acute appendicitis after LA and OA. OA, open appendectomy; LA, laparoscopic appendectomy

For postoperative WBC, NEUT and CPR levels, there were 6, 5 and 7 articles reporting them, respectively. The random-effects model was used due to the significant heterogeneity observed in these studies (I^2^ > 50%, *P* < 0.001). Meta-analysis results showed that patients in the LA group presented significantly reduced postoperative WBC levels [SMD = − 0.432, 95% CI (− 0.775, − 0.089), *P* = 0.013, Fig. 6A], NEUT levels [SMD = − 1.346, 95% CI (− 2.560, − 0.133), *P* = 0.030, Fig. 6B] and CPR levels [SMD = − 2.391, 95% CI (− 3.901, − 0.882), *P* = 0.002, Fig. 6C] compared with the OA group. Since there was significant heterogeneity in the included studies, we conducted sensitivity analyses. The pooled effect size was re-analyzed after removing the included studies one by one, but the new combined results did not change much from the previous ones, suggesting low sensitivity and stability and credibility of the meta-analysis results (Fig. 7A–C).

## Discussion

In this study, the clinical efficacy of LA and OA in the treatment of AA was systematically evaluated. Through searching the relevant databases a total of 16 studies could be included for meta-analysis. As was shown by the results, the incidence of adverse reactions was significantly lower in the LA group compared with the OA group. Then, we went on to investigate the postoperative recovery of patients in both groups and found LA achieved better outcomes in terms of operation time, postoperative hospital stay, intraoperative blood loss and gastrointestinal function recovery time. In addition, laboratory parameters also revealed a significant decrease in WBC levels, NEUT levels and CPR levels in patients in the LA group.

In view of the incidence of adverse reactions, OA group involves greater variety of adverse events, which may be related to higher possibility of class II and III incisions and contamination of surgical field and medical devices during OA. Besides, bacteria discharged with sweat glands were also likely to contaminate the incision [24].

Previous studies have shown that after LA treatment, patients can return to normal activities and diet earlier than those undergoing OA [25]. Surgical cost of LA is higher than OA, but due to its shorter hospital stay, the amount eventually spent by both approaches is almost equivalent [26, 27]. Operative time was decreased instead of prolonged in the LA group in the above two studies; this trend is different from many RCTs or meta-analyses [28] but similar to the results of our study. Operation duration is affected mainly by proficiency in surgical procedures, operation preparation time, and condition of the appendix observed by laparoscopy [29].

In addition, LA surgery uses a trocar to avoid abdominal wall bleeding caused by layer-by-layer process to access the abdominal cavity, and can reduce unnecessary trauma and bleeding during surgery. During LA, the bowel did not leave the abdominal environment, avoiding intestinal adhesions arising from excessive exposure and drying of the bowel. Finally, Atraumatic grasping is employed in LA surgery to prevent repeated lifting and pinching, gauze packing, and stimulation of glove talc to the intestinal serosa [30]; this also explains less amount of intraoperative blood loss and shorter recovery time of gastrointestinal function in the LA group.

In this paper, the WBC level, NEUT level and CPR level of patients in the LA group were significantly lower than those in the OA group. The trauma left by surgery can cause changes in systemic inflammation and immune response, mainly manifested through changes in cytokine levels [31]. It has been reported in related literatures that neutrophil/lymphocyte ratio, inflammatory factors and serum bilirubin are closely linked with the occurrence and development of AA [32, 33]. Stress response and tissue injury resulting from the surgery can compromise the patient’s postoperative immunity and inflammatory response and further affect the patient’s recovery [34]. A myriad of studies have pointed out that LA has a relationship with fewer wound infections compared with that of OA [7], which can be explained by the use of a wound-protective plastic bag for the removal of the inflamed appendix [35].

This study still has some limitation, and more comparable results are required in the future, such as postoperative pain, time to return to normal activities and readmission.

## Conclusions

LA has the advantages of less adverse reactions, shorter operation time and hospital stay, fewer complications, and lower inflammatory response. Hence, it is safe and feasible surgery for AA. The study serves to fill the research gap in the literature discussing optimization of postoperative recovery by LA, and lays a solid theoretical basis for the development of clinical diagnosis and treatment plan for AA.

## Data Availability

The data used to support the findings of this study are available from the corresponding author upon request.
